# Supplementary material for: Proposal of Model for Evaluation of Viral Kinetics of African/Asian/Brazilian—Zika virus Strains (Step Growth Curve) in Trophoblastic Cell Lines
Source: Viruses. 2023 Jun 27;15(7):1446. doi: 10.3390/v15071446 (PMC10386092; doi:10.3390/v15071446)
Supplement: Supplementary file 1 [file viruses-15-01446-s001.zip › Figure S1_Image result of cytopathic effect of viral kinetics.pdf]

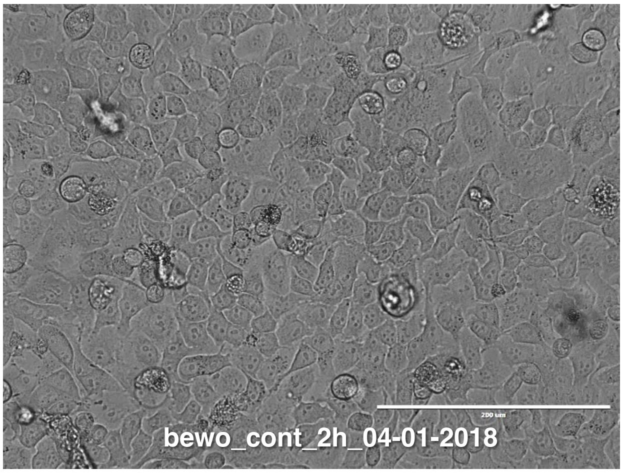

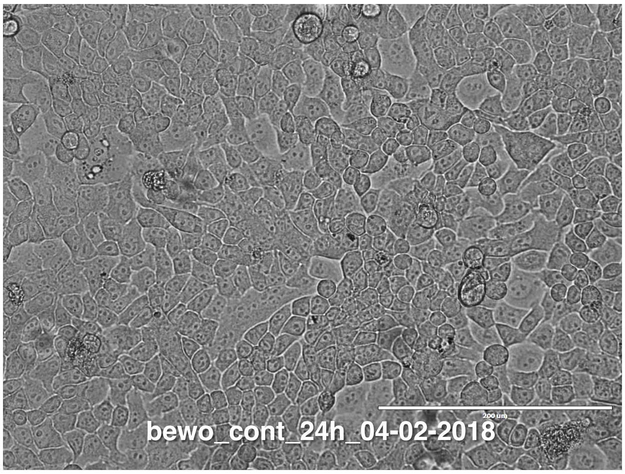

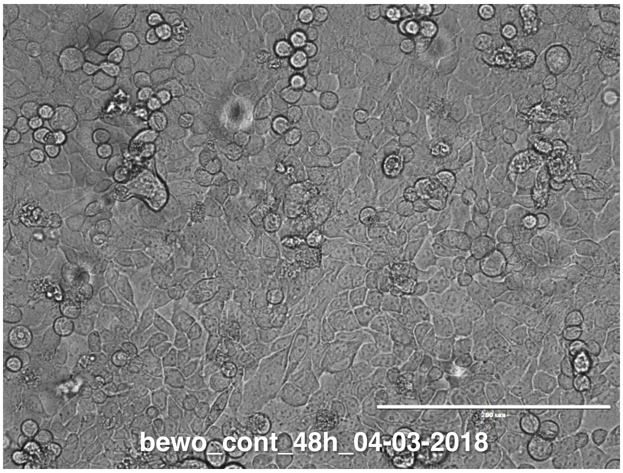

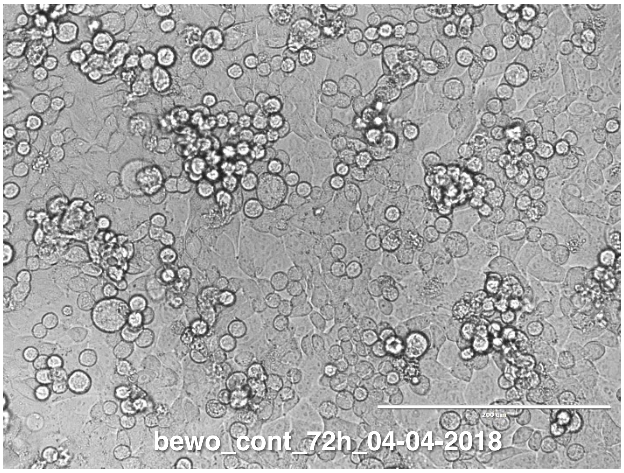

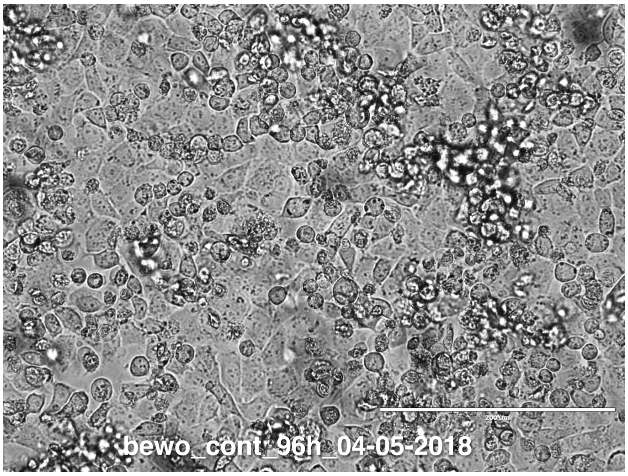

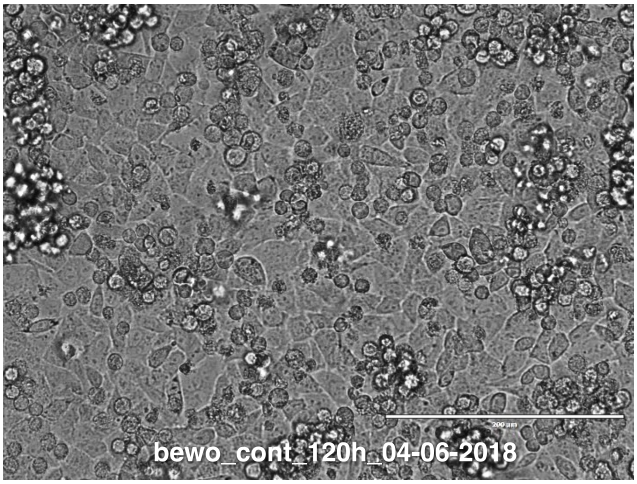

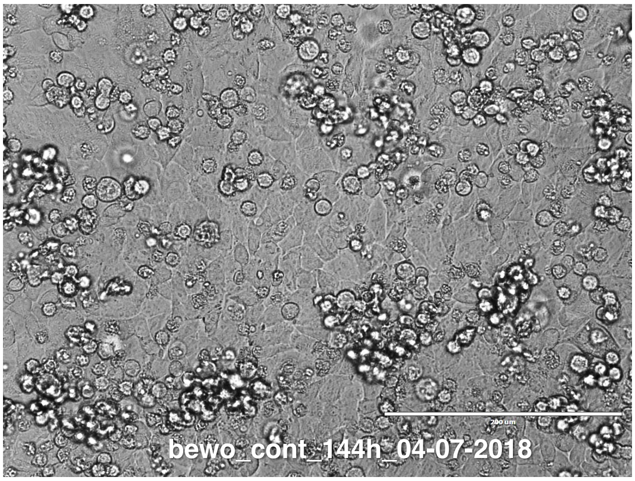

Cellular growth curve of the BeWo cell control. The images show the evolution of cell growth from 2h and then every 24hi until completing 144h. The control follows the hours post infection (hpi) of the cell inoculated with ZIKV-MR766 low passage, an African strain, and ZIKV-IEC-Paraíba, an Asian-Brazilian strain. From the 2 hpi, it is possible to observe the confluence of the cell monolayer with at least 90% coverage. In later hpi, the rounded, refractive cells present in the mat overlay are dead. The cell monolayer stays together until the last hpi.

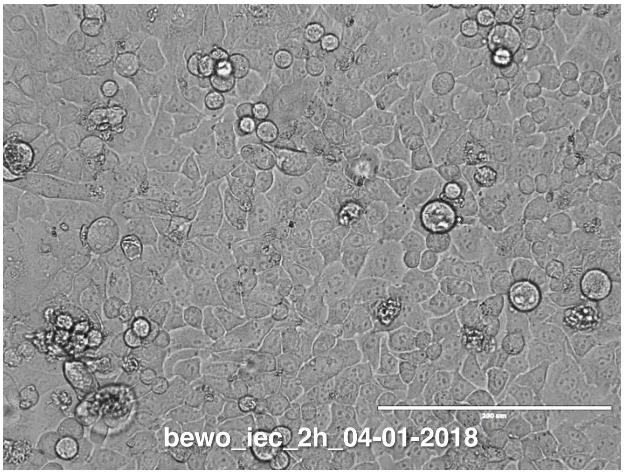

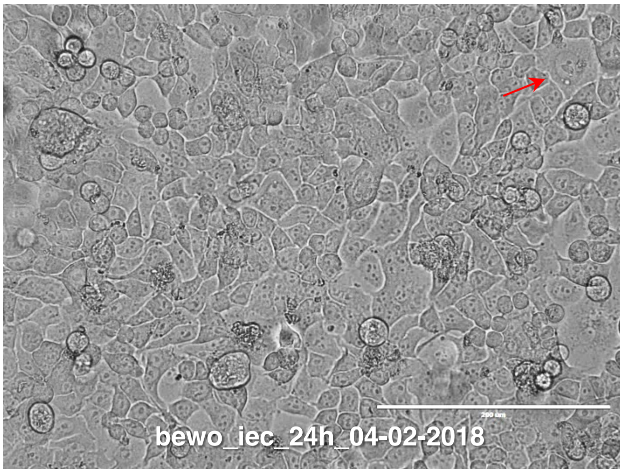

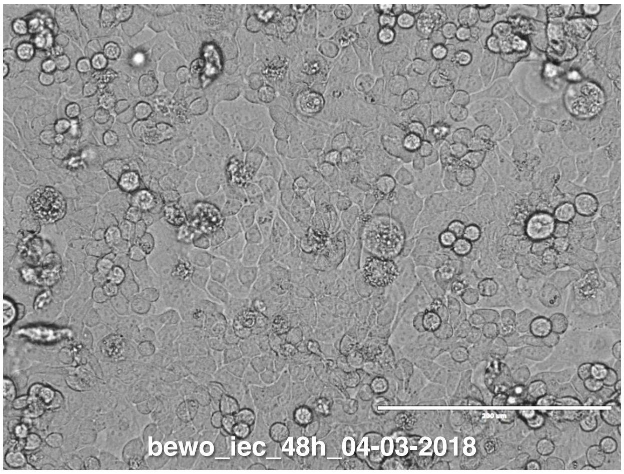

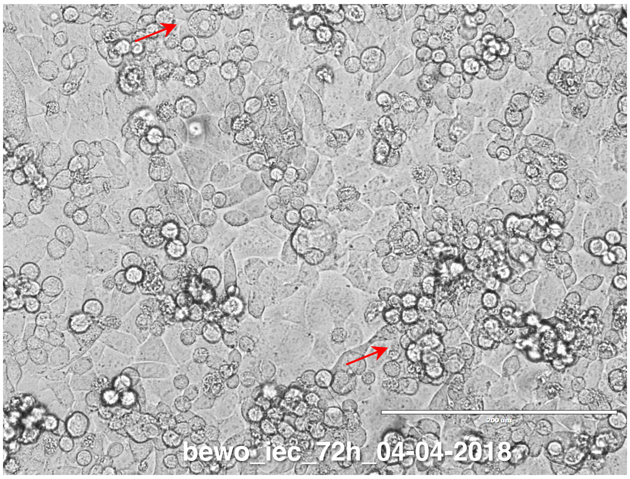

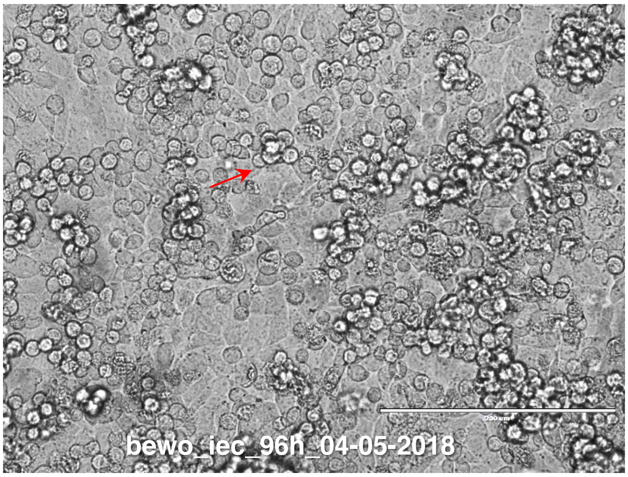

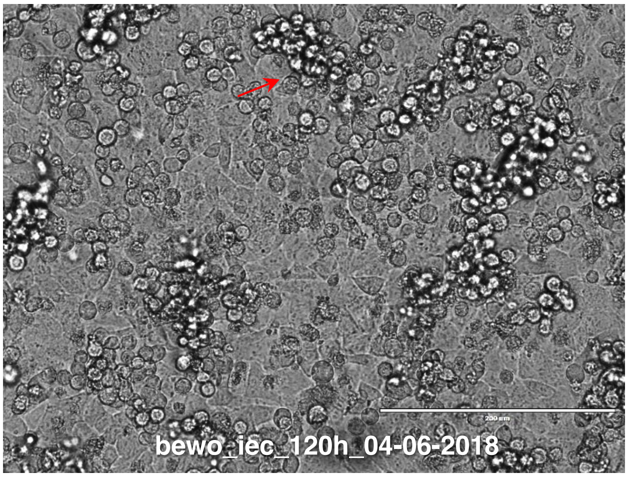

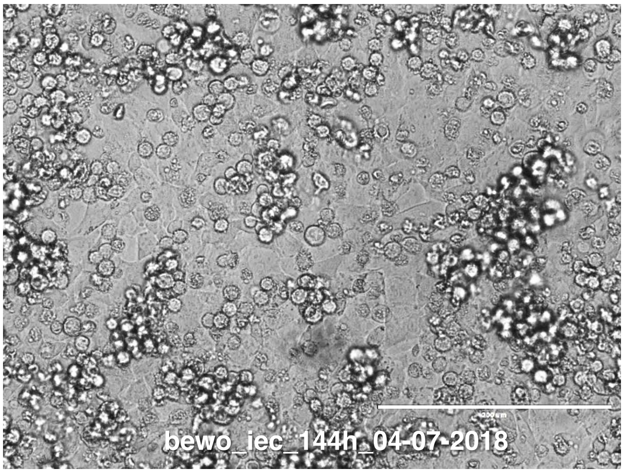

Viral kinetics of BeWo inoculated with ZIKV-IEC-Paraíba. The images show the inoculation from 2h and then every 24h until completing 144h. The kinetics follows the hours post infection (hpi) of the inoculated cell. The red arrow points to the observed cytopathic effect. From the 2h hpi it is possible to observe the confluence of the cell monolayer with at least 90% coverage and no evidence of infection. From the 24h hpi, there are signs of cytopathic effect. In later hpi there are dead cells (rounded and refractive) present in the supernatant and cells with signs of degeneration in the cell tapetum. The cell monolayer stays together until the last hpi.

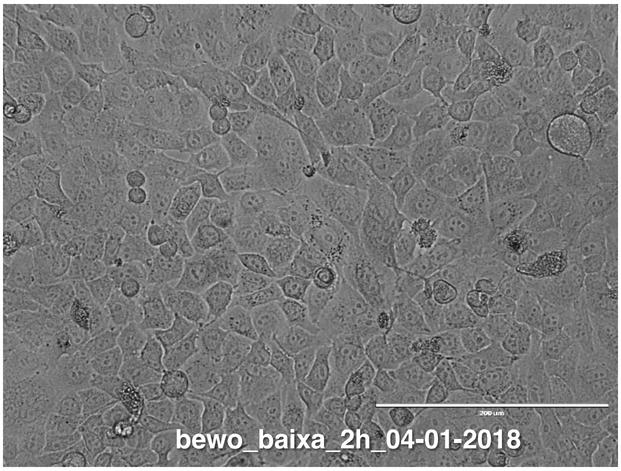

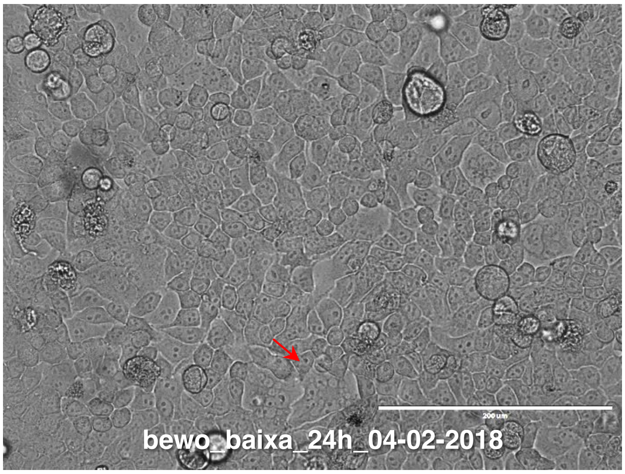

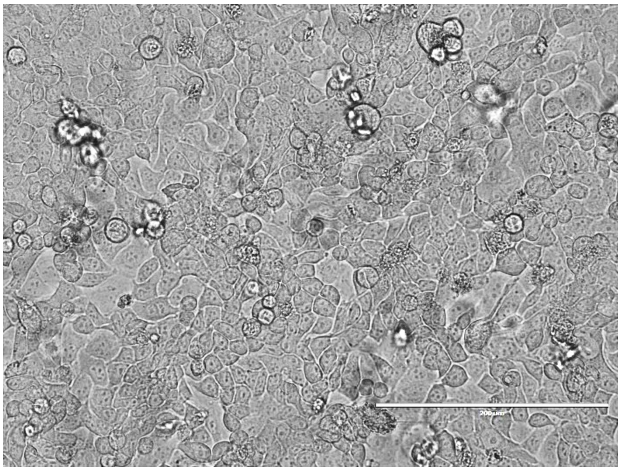

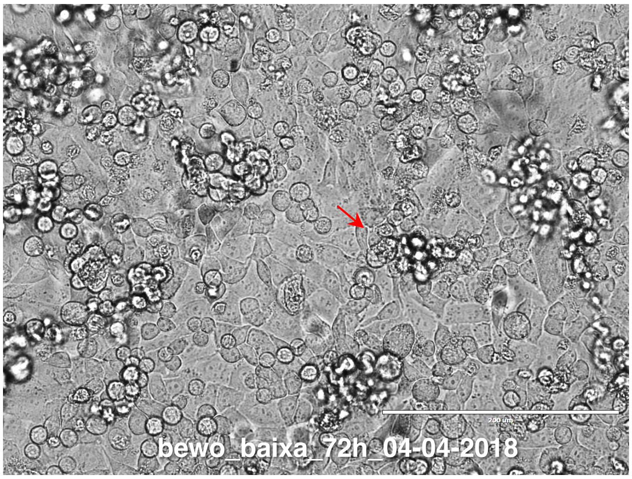

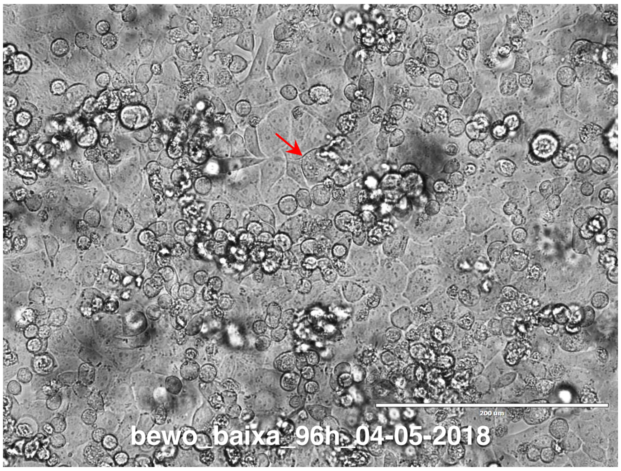

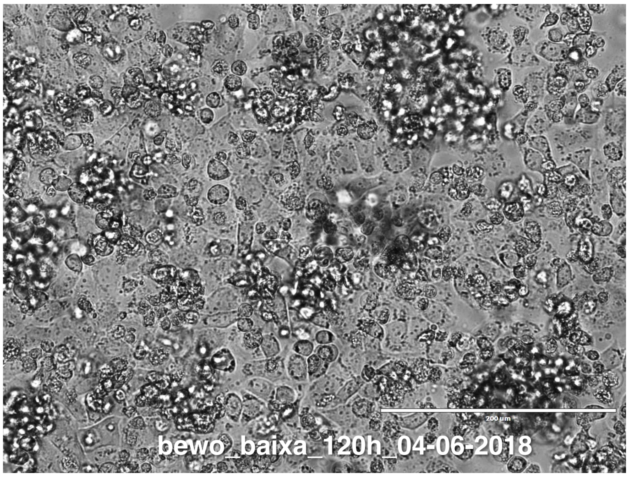

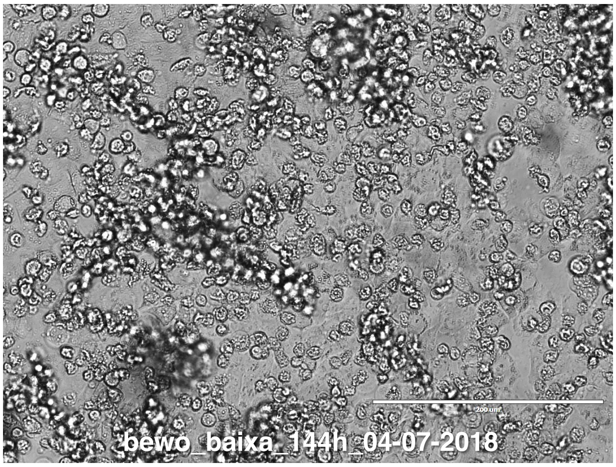

Viral kinetics of BeWo inoculated with ZIKV-MR766bp. The images show the inoculation starting at 2h and then every 24h until completing 144h. The kinetics follow the hours post-infection (hpi) of the inoculated cell. The red arrow points to the observed cytopathic effect. From the 2 hpi, it is possible to observe the confluence of the cell monolayer with at least 90% coverage and no evidence of infection. From the 24 hpi, there are signs of a cytopathic effect. In later hpi, there are dead cells (rounded and refractive) present in the supernatant and cells with signs of degeneration in the cell tapetum. The cell monolayer shows progressive degeneration at 96 hpi.

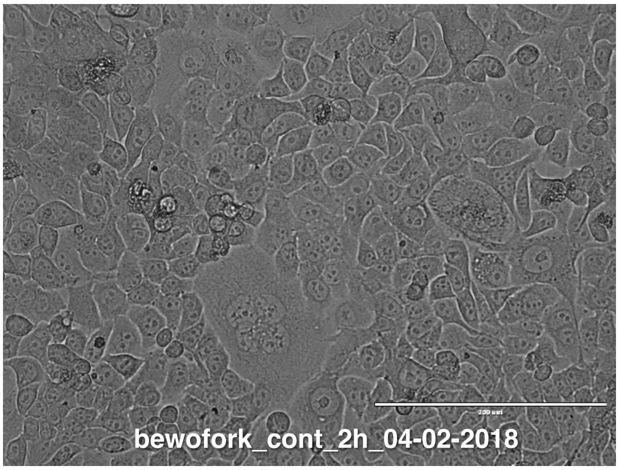

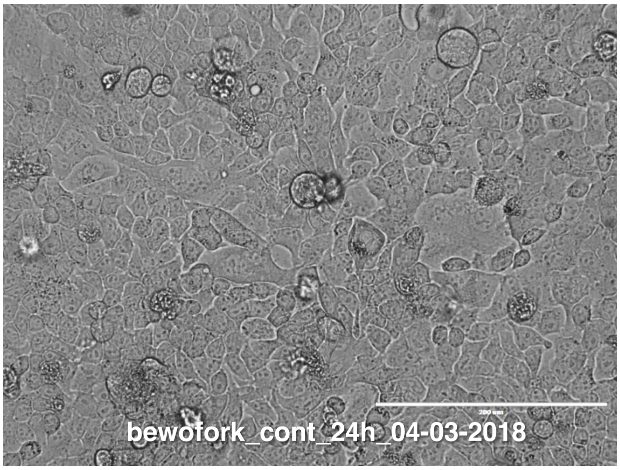

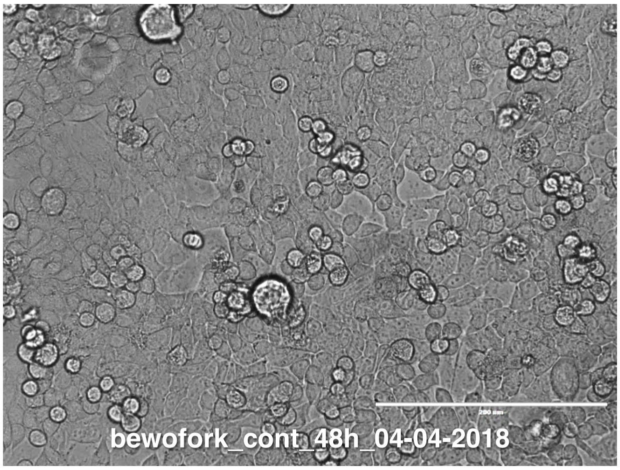

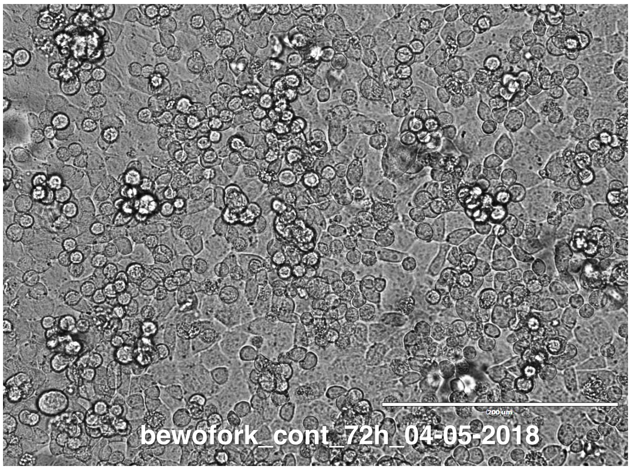

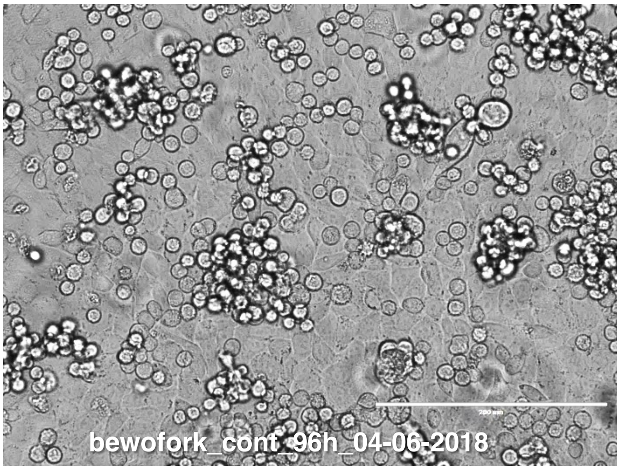

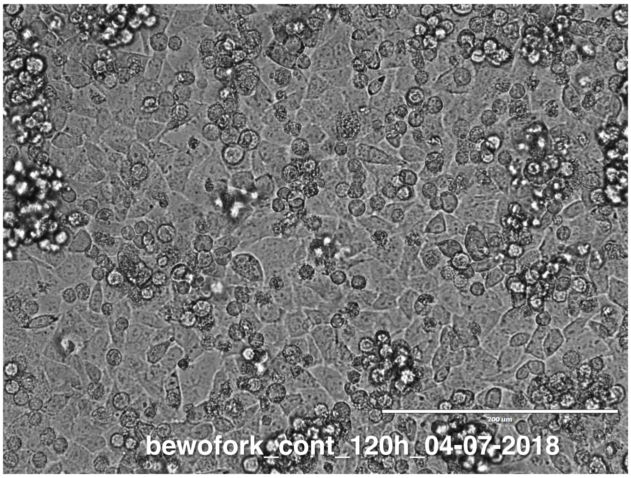

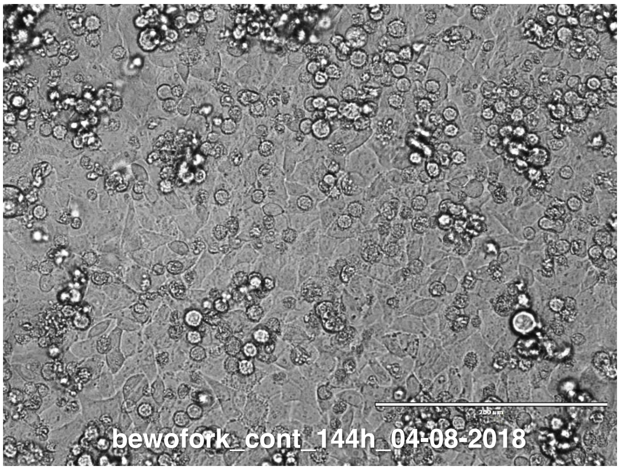

Cell growth curve of the BeWo cell treated with forskolin and control. The images show the evolution of cell growth from 2h and then every 24h until completing 144h. The control follows the hours post infection (hpi) of the cell inoculated with ZIKV-MR766 low passage, an African strain, and ZIKV-IEC-Paraíba, Asian-Brazilian strain. From the 2 hpi, it is possible to observe the confluence of the cell monolayer with at least 90% coverage. In later hpi, the rounded, refractive cells present in the mat overlay are dead. The cell monolayer stays together until the last hpi.

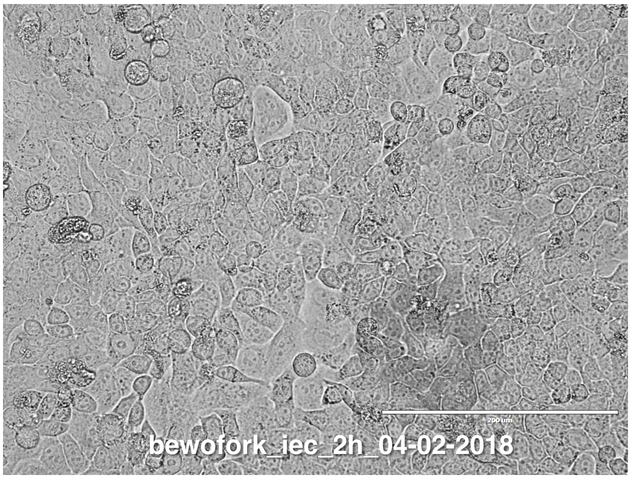

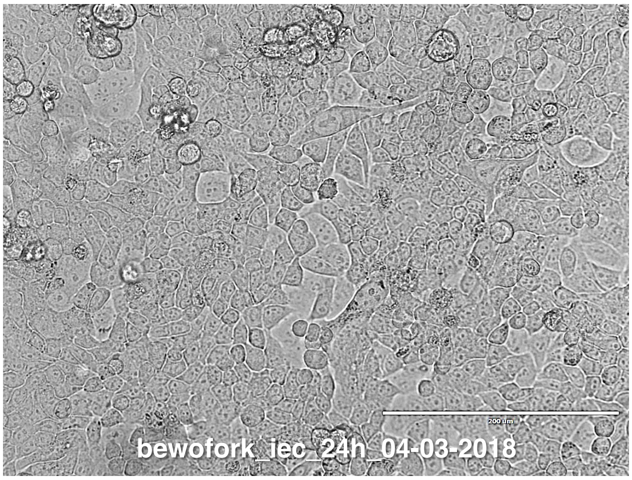

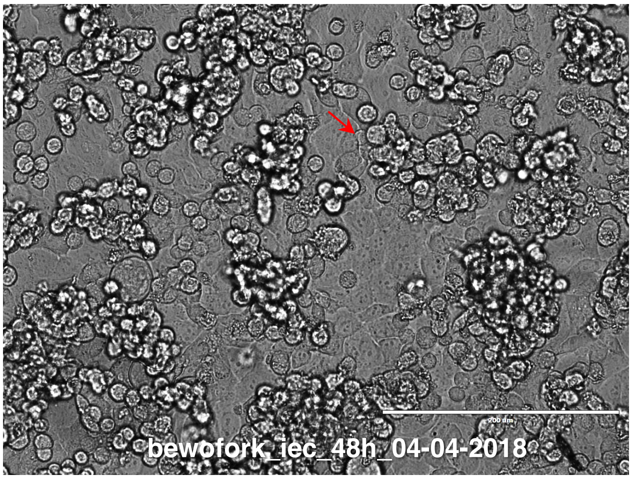

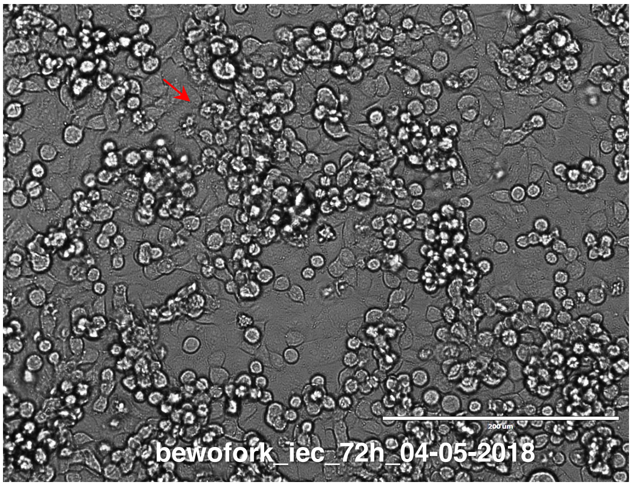

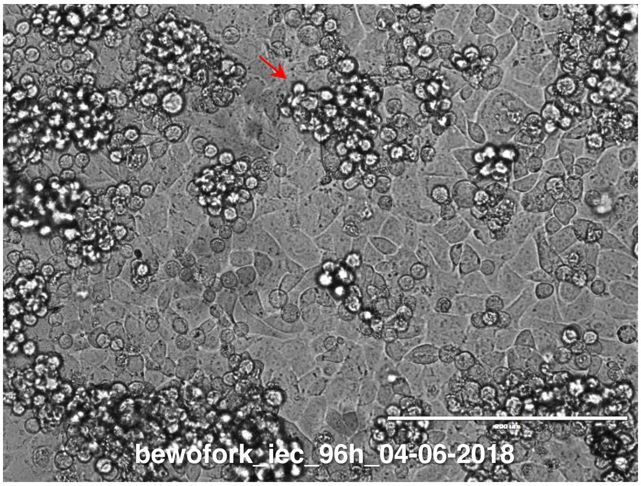

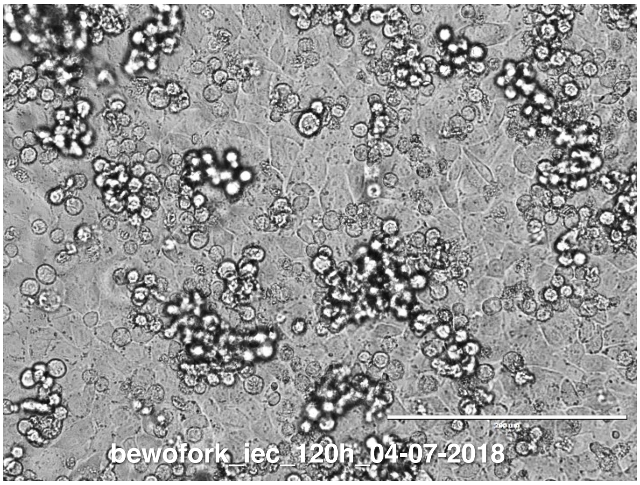

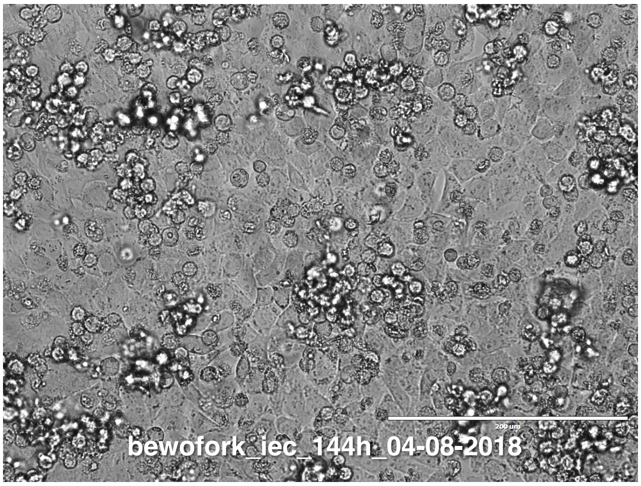

Viral kinetics of BeWo with forskolin inoculated with ZIKV-IEC-Paraíba. The images show the inoculation beginning at 2h and progressing every 24h until it reaches 144h. The kinetics follow the hours post-infection (hpi) of the inoculated cell. The red arrow points to the observed cytotoxic effect. From the 2 hpi, it is possible to observe the confluence of the cell monolayer with at least 90% coverage and no evidence of infection. From the 24 hpi, there are signs of a cytopathic effect. In later hpi, there are dead cells (rounded and refractive) present in the supernatant and cells with signs of degeneration in the cell monolayer. The cell monolayer stays together until the last hpi.

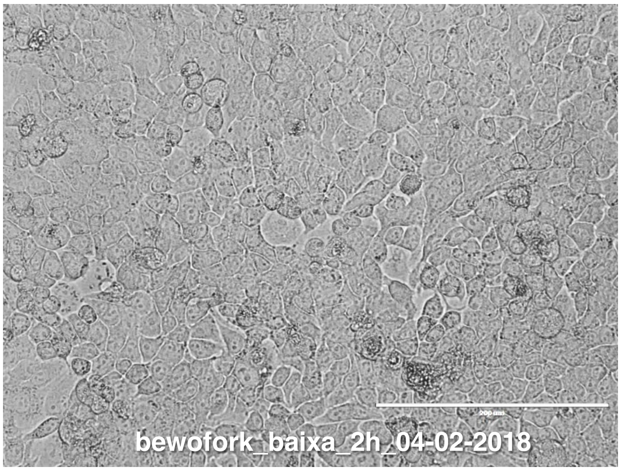

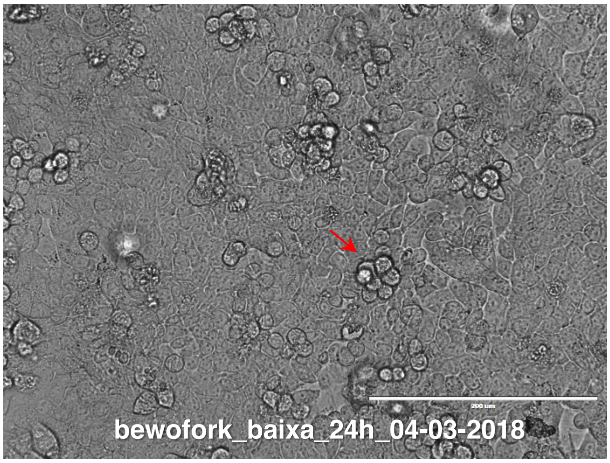

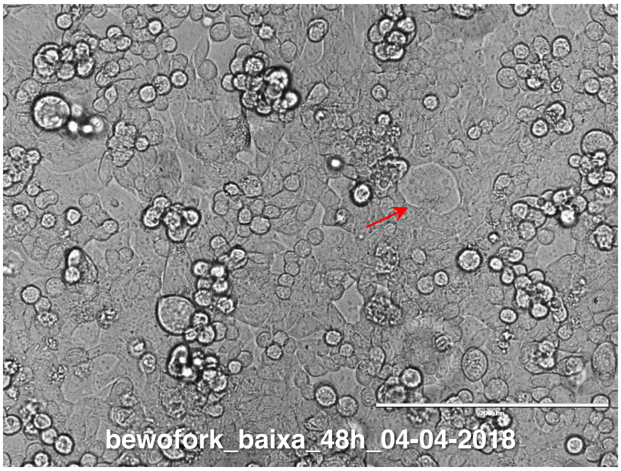

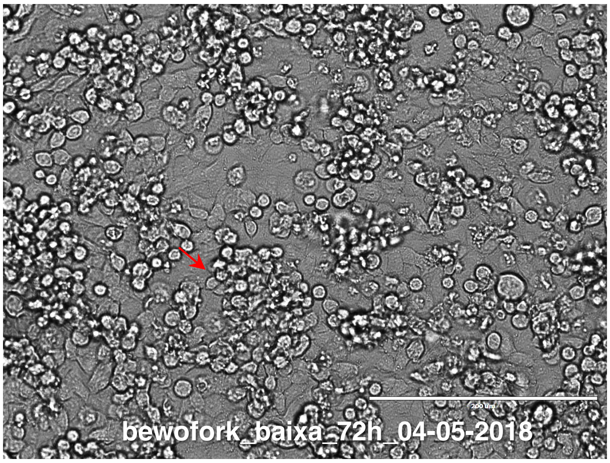

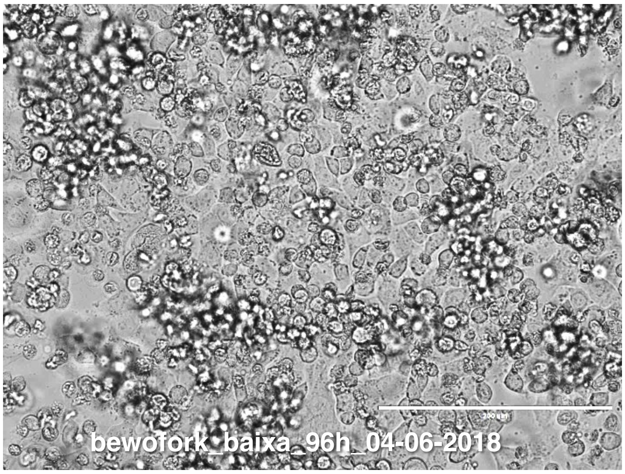

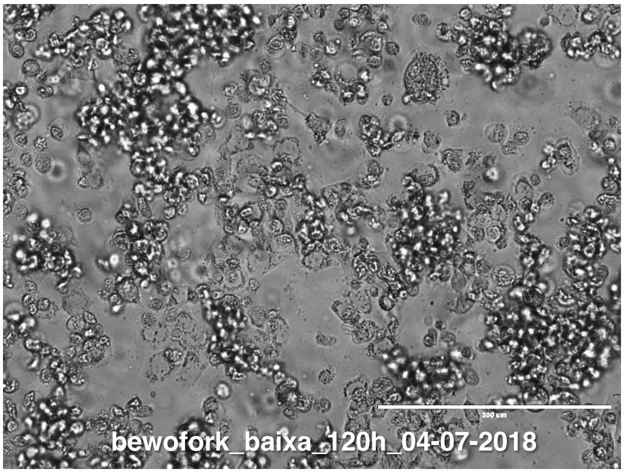

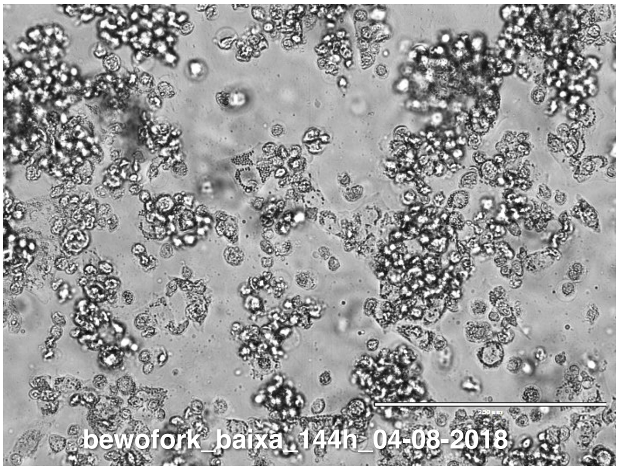

Viral kinetics of BeWo with forskolin inoculated with ZIKV-MR766bp The images show the inoculation from 2h and then every 24h until completing 144h. The kinetics follow the hours post infection (hpi) of the inoculated cell. The red arrow points to the observed cytopathic effect. From the 2 hpi, it is possible to observe the confluence of the cell monolayer with at least 90% coverage and no evidence of infection. At 24 hpi, there are dead cells (rounded and refractive) present in the supernatant. From 96 onward, there is cellular monolayer degeneration and accumulation of cellular debris. At 144 hpi, virtually all cells are dead.

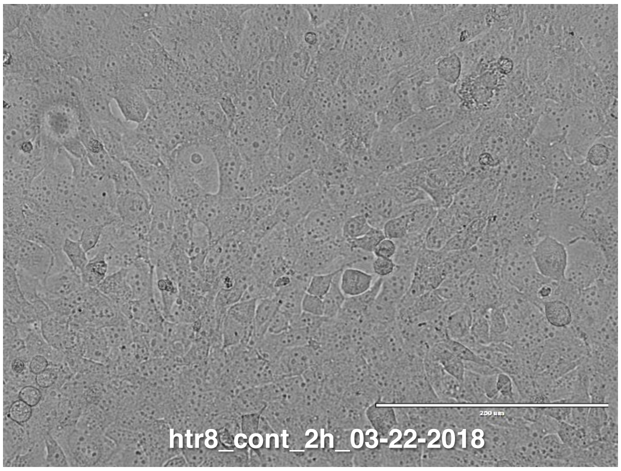

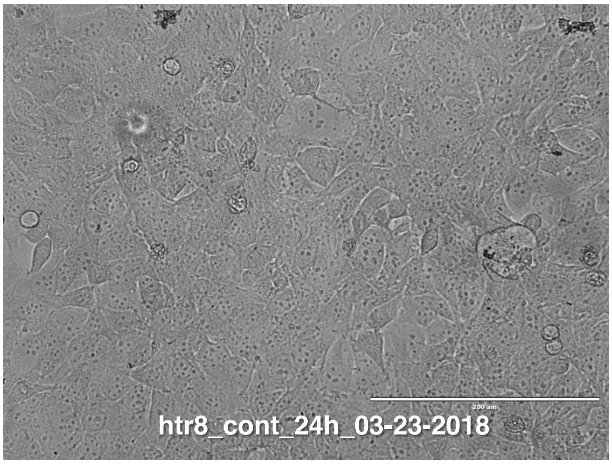

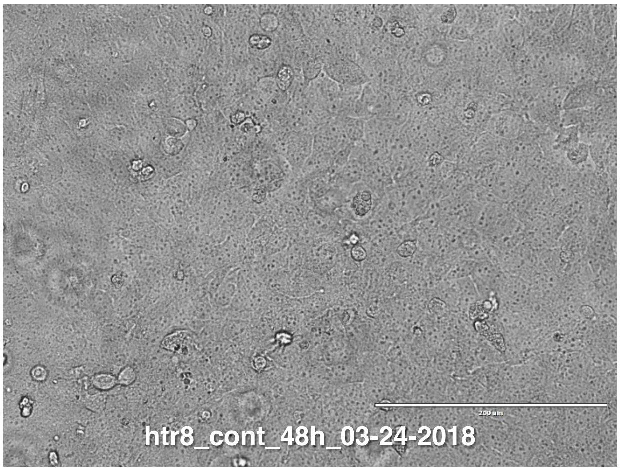

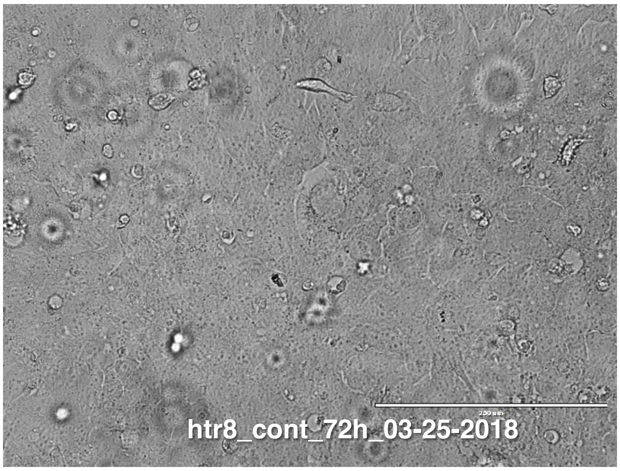

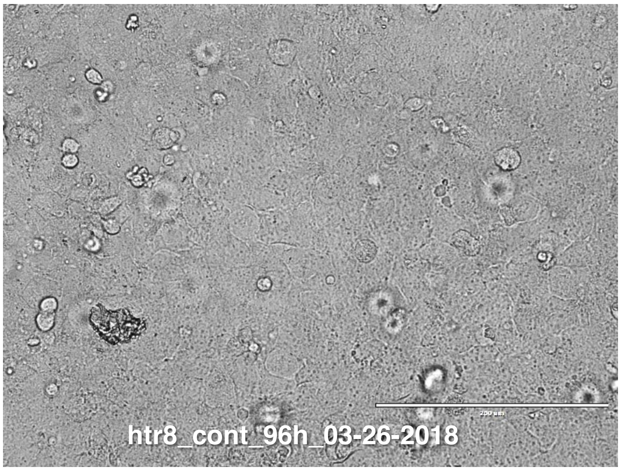

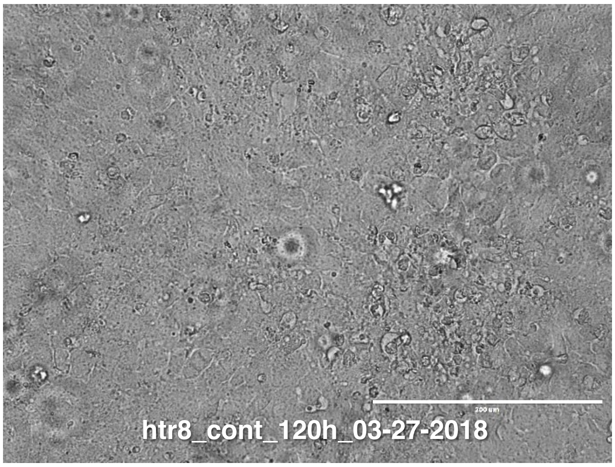

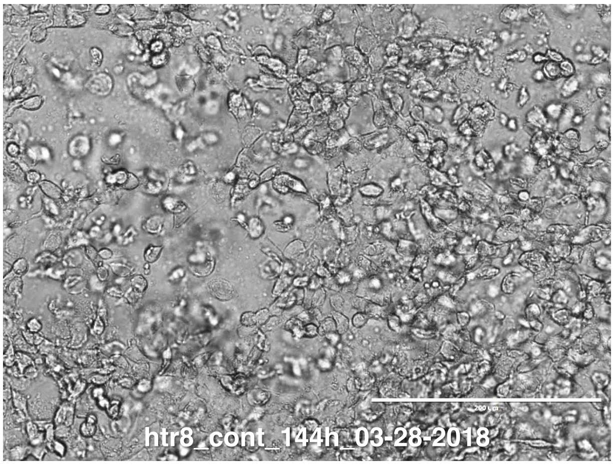

Cell growth curve of HTR-8 cells, control. The images show the evolution of cell growth from 2h and then every 24h until completing 144h. The control follows the hours post infection (hpi) of the cell inoculated with ZIKV-MR766 low passage, an African strain, and ZIKV-IEC-Paraíba, an Asian-Brazilian strain. From the 2 hpi, it is possible to observe the confluence of the cell monolayer with at least 90% coverage. Up to 120 hpi, the monolayer remains intact. At 144 hpi, there are large amounts of dead cells in the supernatant, and the cell monolayer remains partially intact.

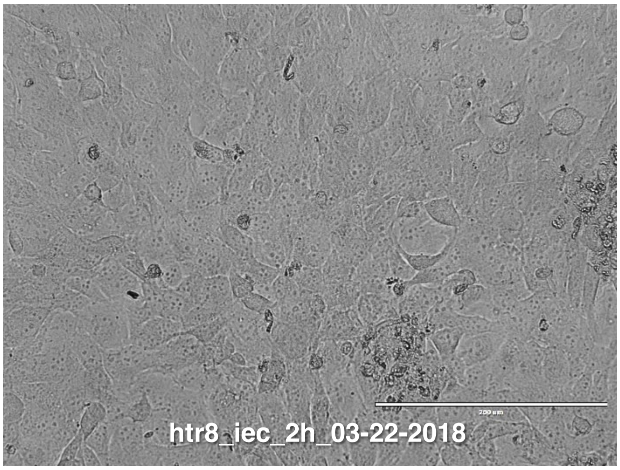

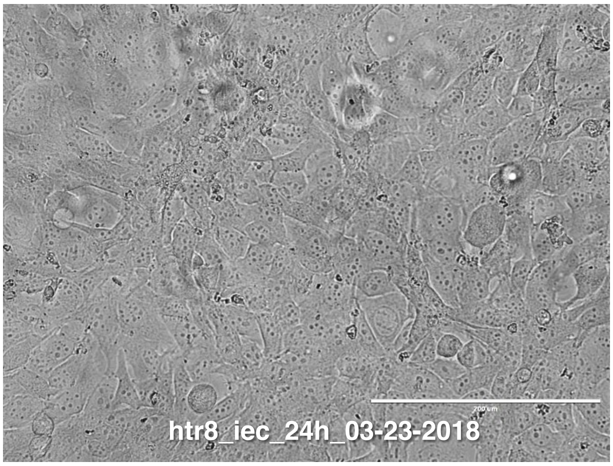

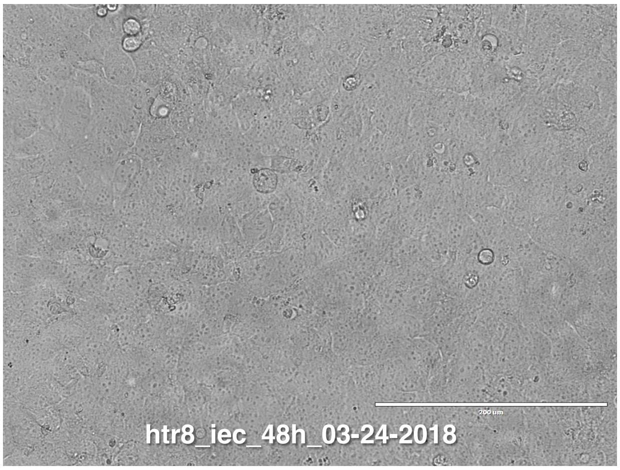

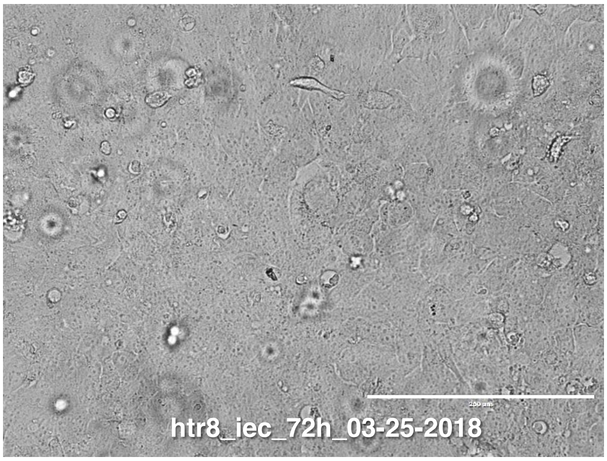

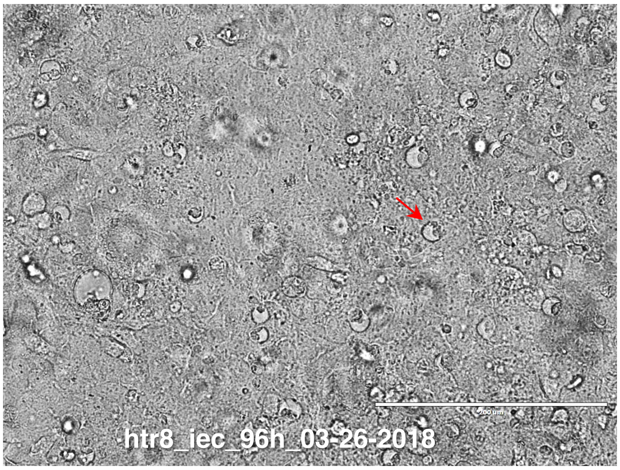

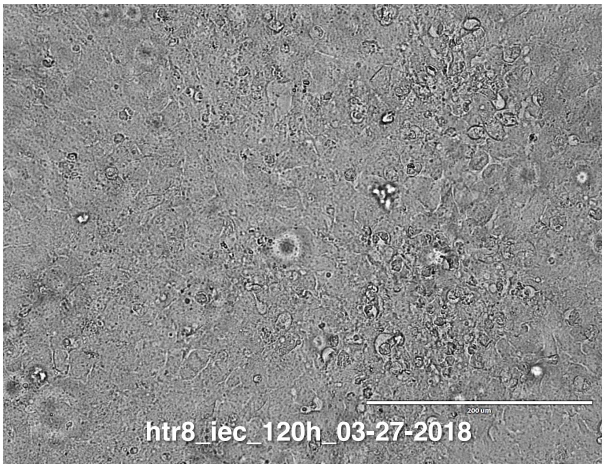

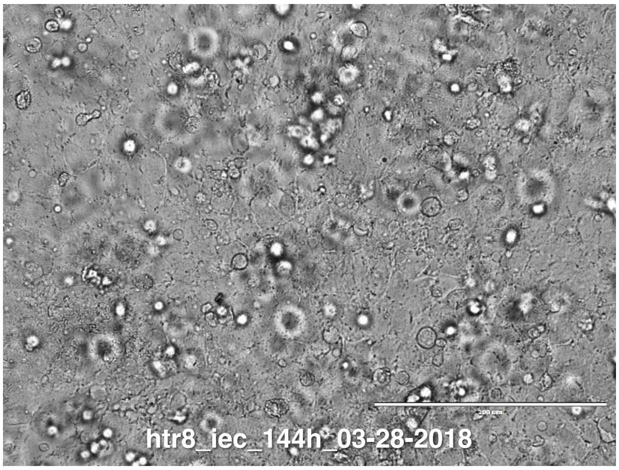

Viral kinetics of HTR-8 inoculated with ZIKV-IEC-Paraíba. The images show the inoculation starting at 2h and then every 24h until completing 144h. The kinetics follow the hours post infection (hpi) of the inoculated cell. The red arrow points to the observed cytopathic effect. From the 2 hpi, it is possible to observe the confluence of the cell monolayer with at least 90% coverage and no evidence of infection, which continues until the 72 hpi. From 96 to 144 hpi, there are few signs of a cytopathic effect. The cell monolayer stays together for all hpi.

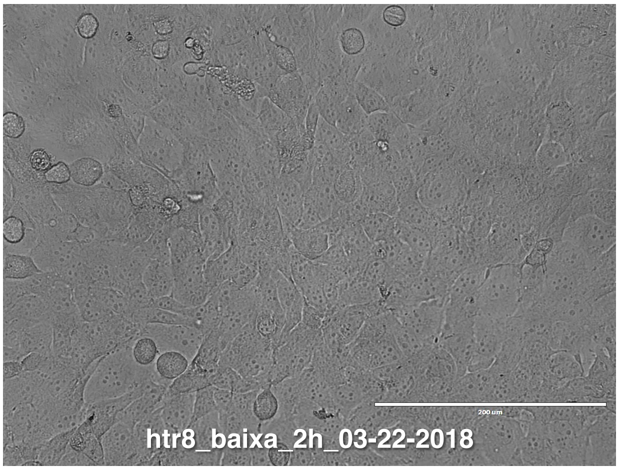

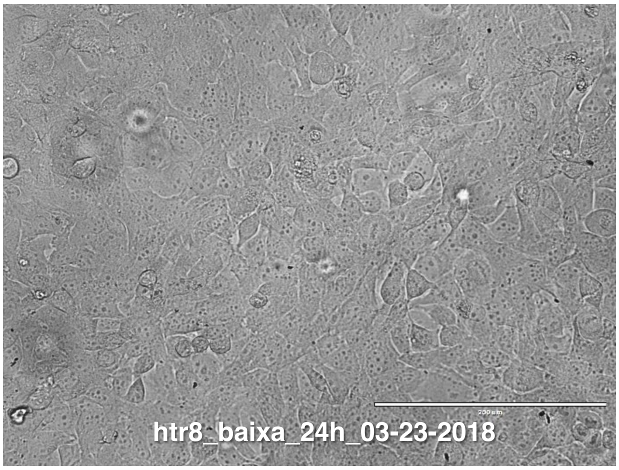

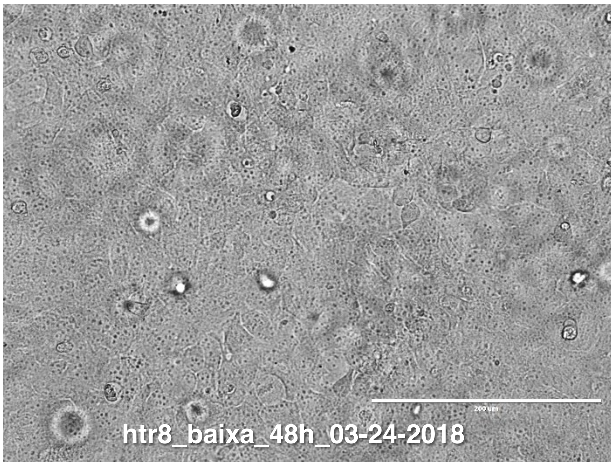

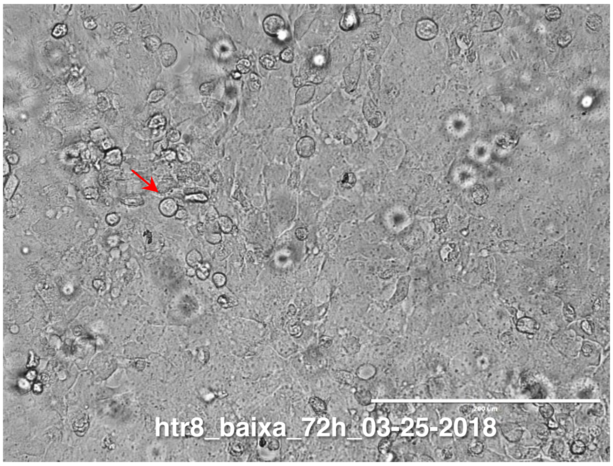

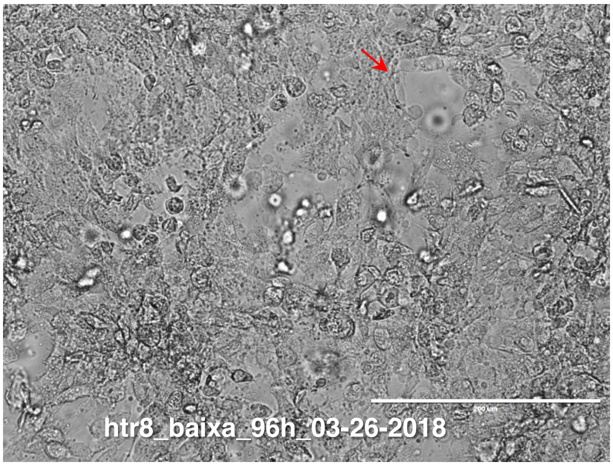

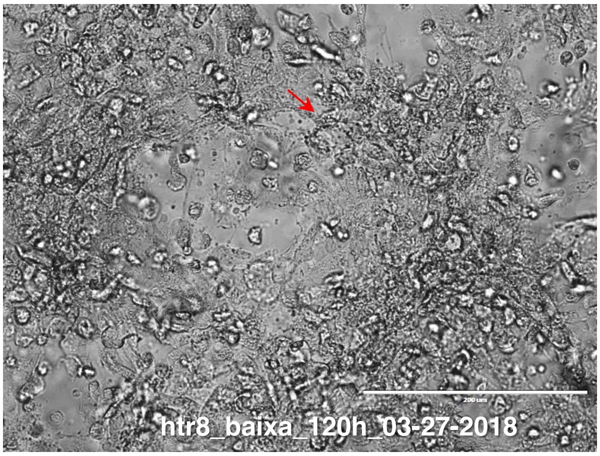

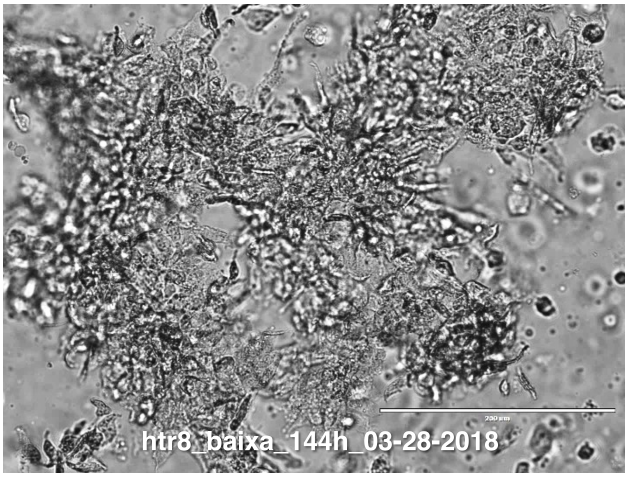

Viral kinetics of HTR-8 inoculated with ZIKV-MR766lp. The images show the inoculation starting at 2h and then every 24h until completing 144h. The kinetics follow the hours post infection (hpi) of the inoculated cell. The red arrow points to the observed cytopathic effect. From the 2 hpi, it is possible to observe the confluence of the cell monolayer with at least 90% coverage and no evidence of infection. Up to 48 hpi, there are no signs of infection. After 72 hpi, dead cells (rounded and refractive) are observed in the supernatant. From 96 hpi onward, there is an accelerated cell monolayer degeneration and accumulation of cell debris in the supernatant. At 144 hpi, virtually all cells are dead.

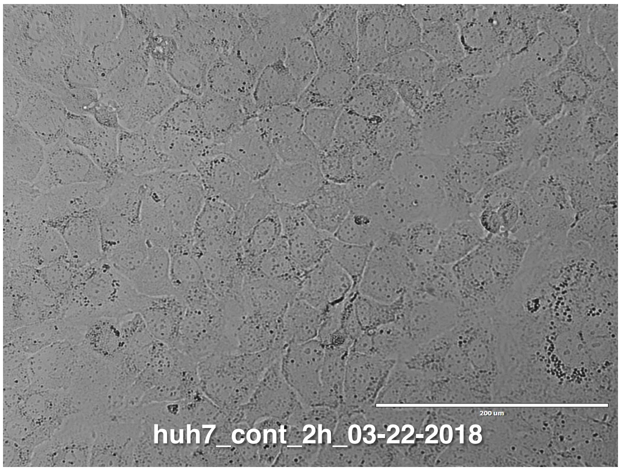

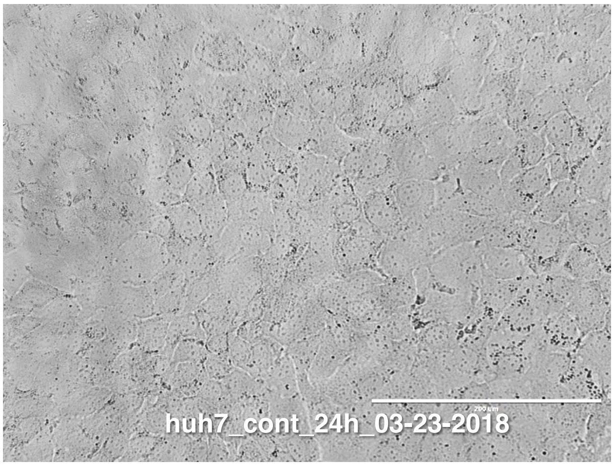

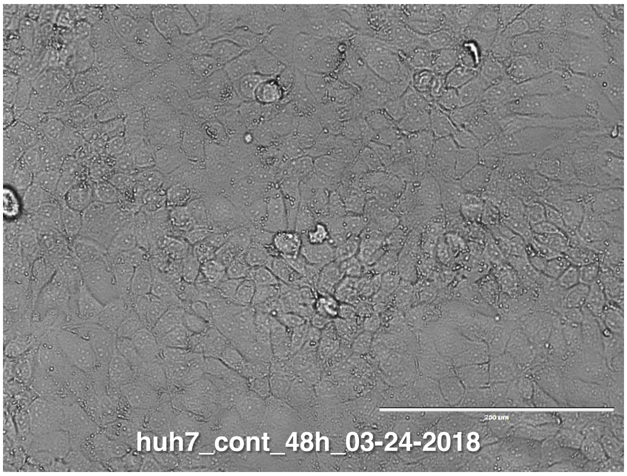

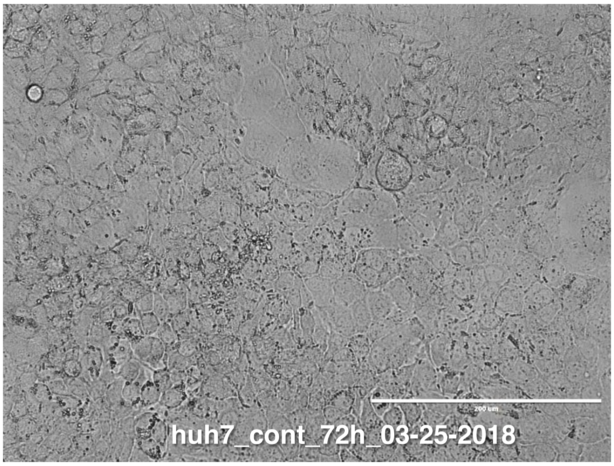

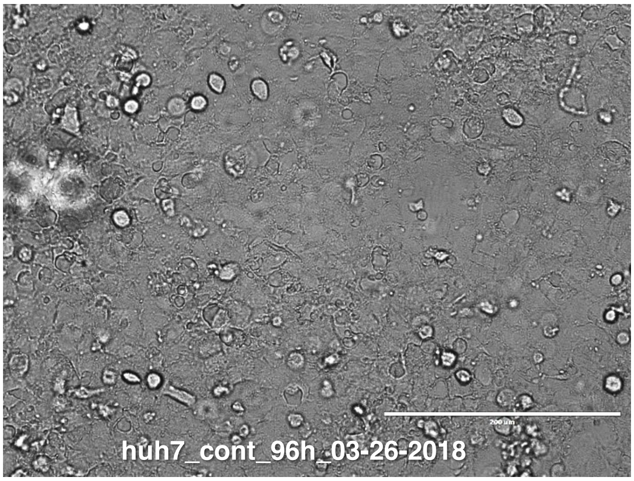

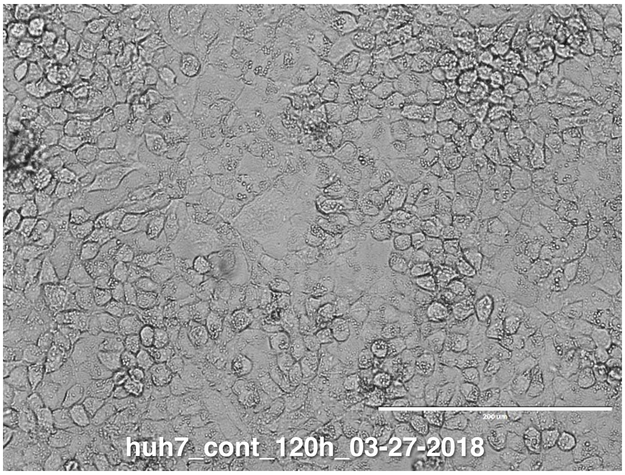

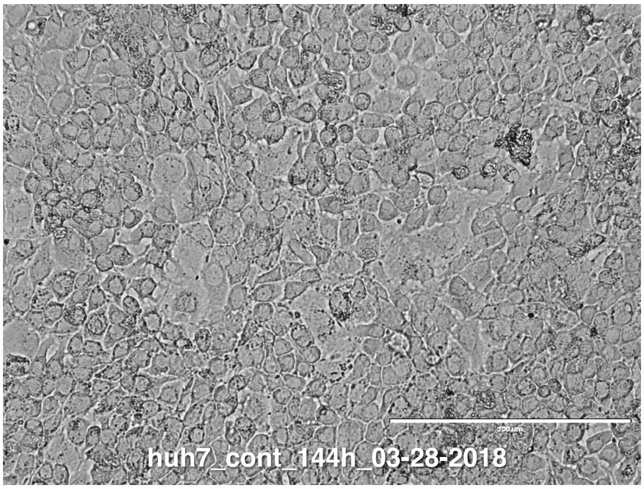

Cellular growth curve of the HuH-7 cell control. The images show the evolution of cell growth from 2h and then every 24h until completing 144h. The control follows the hours-post-infection (hpi) of the cell inoculated with ZIKV-MR766 low passage, an African strain, and ZIKV-IEC-Paraíba, an Asian-Brazilian strain. From the 2 hpi it is possible to observe the confluence of the cell monolayer with at least 90% coverage. The cell monolayer is maintained up to 144 hpi with little cell death.

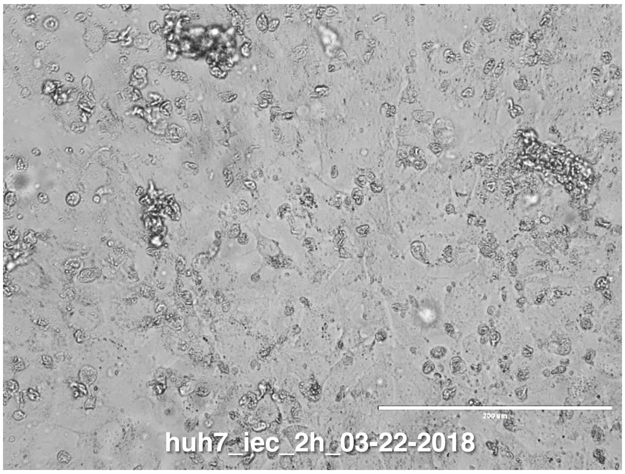

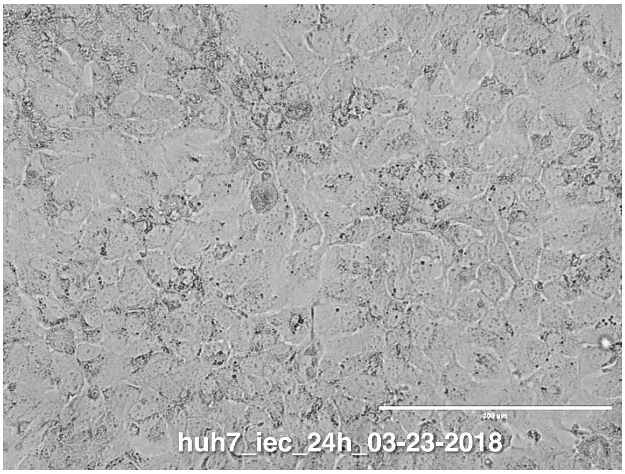

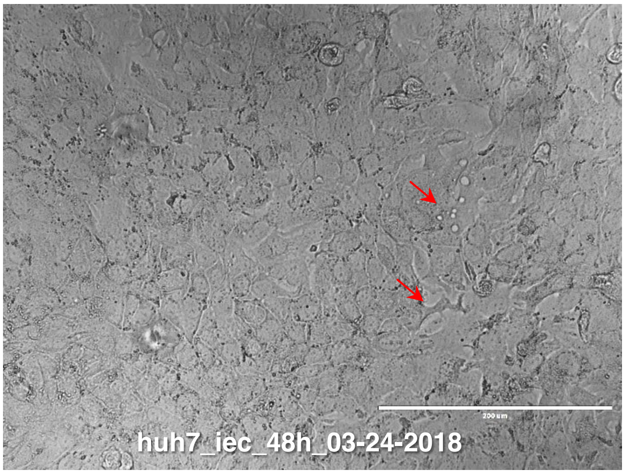

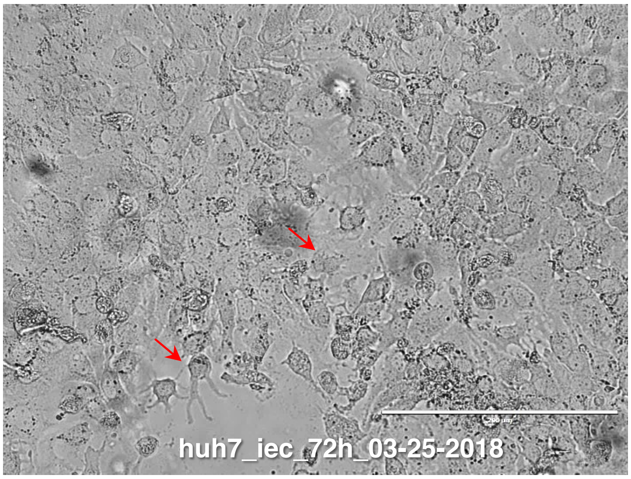

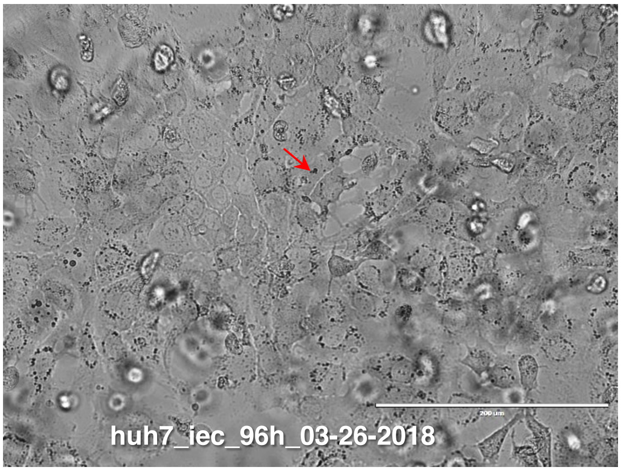

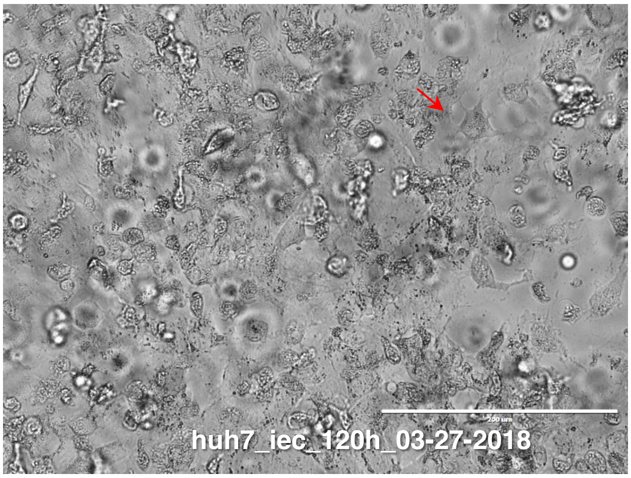

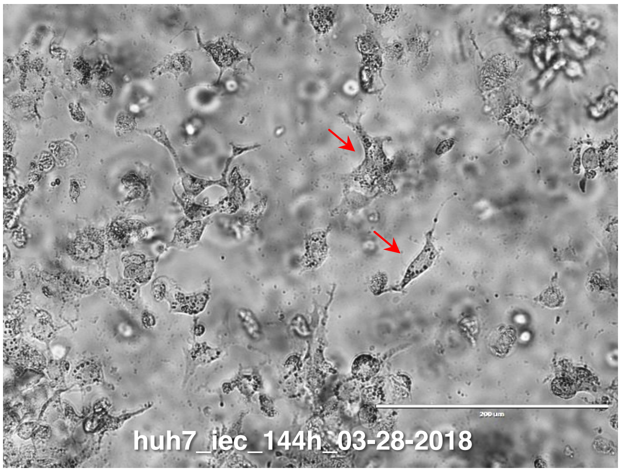

Viral kinetics of HuH-7 inoculated with ZIKV-IEC-Paraíba. The images show the inoculation starting at 2 h and then every 24h until completing 144h. The kinetics follow the hours post infection (hpi) of the inoculated cell. The red arrow points to the observed cytopathic effect. From the 2 hpi, it is possible to observe the confluence of the cell monolayer with at least 90% coverage. At 48 hpi, it shows signs of a cytotoxic effect. From 72 to 144 hpi, the signs intensify, such as the degeneration of the cell monolayer and cell debris in the supernatant. At 144 hpi, practically all cells are lysed.

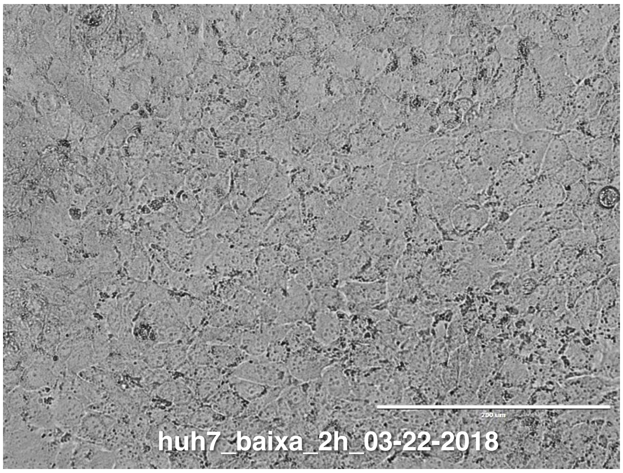

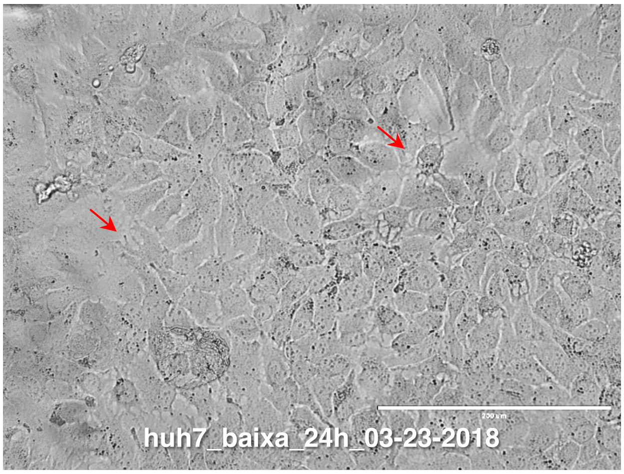

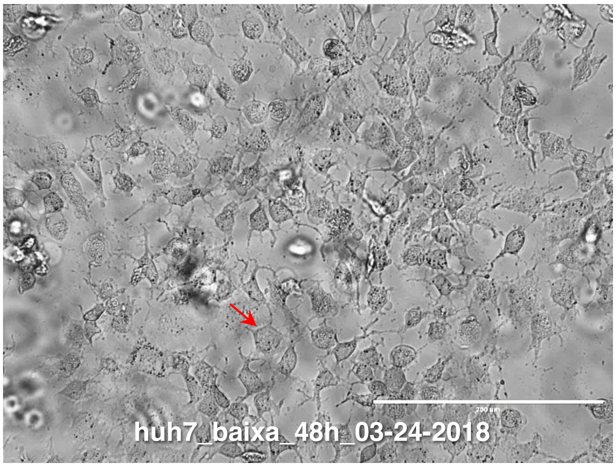

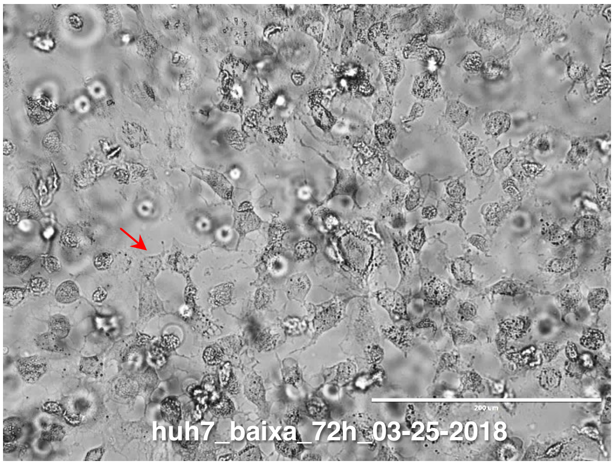

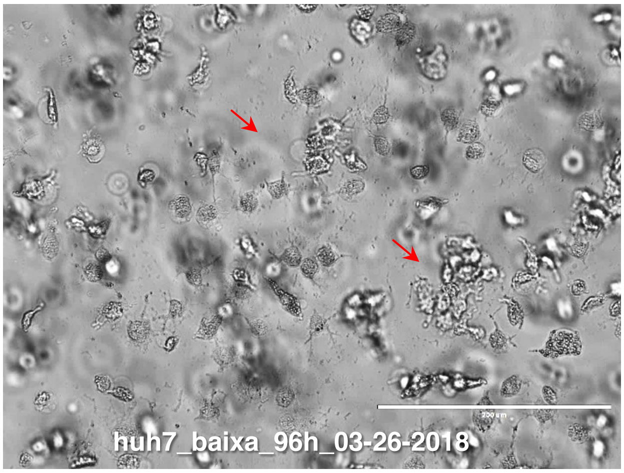

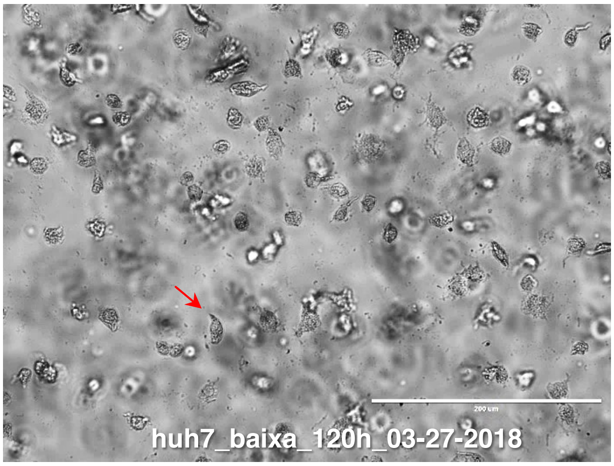

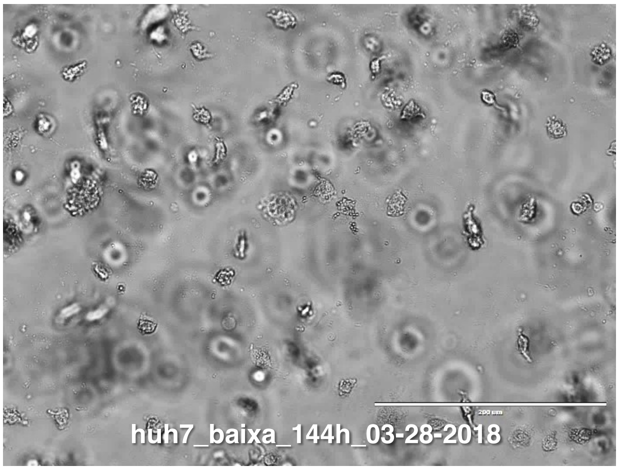

Viral kinetics of HuH-7 inoculated with ZIKV-MR766bp. The images show the inoculation from 2h and then every 24h until completing 144h. The kinetics follow the hours post infection (hpi) of the inoculated cell. The red arrow points to the observed cytopathic effect. From the 2 hpi, it is possible to observe the confluence of the cell monolayer with at least 90% coverage and no evidence of infection. At 24 hpi, the first signs of a cytopathic effect are observed. At 48 and 72 hpi, most of the cells are infected, and part of the cell mat has detached due to cell lysis. At 96 hpi, all cells are dead; there is an accumulation of cell debris in the supernatant and some dead cells that remained attached to the surface of the flask.
